# Cost-utility analysis of TAVI compared with surgery in patients with severe aortic stenosis at low risk of surgical mortality in the Netherlands

**DOI:** 10.1186/s12962-024-00531-6

**Published:** 2024-03-26

**Authors:** Rob Eerdekens, Suzanne Kats, Janneke PC Grutters, Michelle Green, Judith Shore, Pascal Candolfi, Wija Oortwijn, Pim Van Der Harst, Pim Tonino

**Affiliations:** 1 Heart Center, Catharina Hospital, Eindhoven, The Netherlands; 2 Maastricht University Medical Center, Maastricht, The Netherlands; 3 Radboud University Medical Center, Nijmegen, The Netherlands; 4 York Health Economics Consortium, University of York, Heslington, York, UK; 5 Edwards Lifesciences, Nyon, Switzerland; 6 University Medical Center, Utrecht, The Netherlands

**Keywords:** Transcatheter aortic valve replacement, Surgery, Heart, Cost-utility analysis, Aortic valve stenosis, Risk

## Abstract

**Background:**

There is growing evidence to support the benefits of transcatheter aortic valve implantation (TAVI) over surgical aortic valve replacement (SAVR) in patients with symptomatic severe aortic stenosis (sSAS) who are at high- or intermediate-risk of surgical mortality. The PARTNER 3 trial showed clinical benefits with SAPIEN 3 TAVI compared with SAVR in patients at low risk of surgical mortality. Whether TAVI is also cost-effective compared with SAVR for low-risk patients in the Dutch healthcare system remains uncertain. This article presents an analysis using PARTNER 3 outcomes and costs data from the Netherlands to inform a cost-utility model and examine cost implications of TAVI over SAVR in a Dutch low-risk population.

**Methods:**

A two-stage cost-utility analysis was performed using a published and validated health economic model based on adverse events with both TAVI and SAVR interventions from a published randomized low risk trial dataset, and a Markov model that captured lifetime healthcare costs and patient outcomes post-intervention. The model was adapted using Netherlands-specific cost data to assess the cost-effectiveness of TAVI and SAVR. Uncertainty was addressed using deterministic and probabilistic sensitivity analyses.

**Results:**

TAVI generated 0.89 additional quality-adjusted life years (QALYs) at a €4742 increase in costs per patient compared with SAVR over a lifetime time horizon, representing an incremental cost-effectiveness ratio (ICER) of €5346 per QALY gained. Sensitivity analyses confirm robust results, with TAVI remaining cost-effective across several sensitivity analyses.

**Conclusions:**

Based on the model results, compared with SAVR, TAVI with SAPIEN 3 appears cost-effective for the treatment of Dutch patients with sSAS who are at low risk of surgical mortality. Qualitative data suggest broader societal benefits are likely and these findings could be used to optimize appropriate intervention selection for this patient population.

**Supplementary Information:**

The online version contains supplementary material available at 10.1186/s12962-024-00531-6.

## Introduction

Surgical aortic valve replacement (SAVR) is an established option for severe aortic stenosis, with real-world evidence supporting its benefits in patients across a range of risk levels of surgical mortality [1]. For patients who are not suitable for surgery, transcatheter aortic valve implantation (TAVI) has emerged as the treatment of choice [2]. As TAVI has evolved, with accumulation of clinical experience and simplification of the procedure, together with improvements in valve design and delivery, there has been a reduction in complication rates and a move towards its use in lower risk patient populations [3].

Since the first TAVI procedure 20 years ago, multiple clinical registries and trials have established this intervention as a valuable treatment for symptomatic severe aortic stenosis (sSAS) [4]. The PARTNER 3 study showed that SAPIEN 3 TAVI reduced a composite endpoint of death, stroke, or rehospitalization after 1 and 2 years, versus SAVR [5, 6], and revealed an improvement in efficacy over previous versions of the device used in both the original PARTNER trial (SAPIEN valve) in patients ineligible for surgery or at high risk of mortality and in PARTNER 2 (SAPIEN-XT valve) in patients at intermediate risk [7]. Prior to the PARTNER 3 and Evolut Low Risk trials [6, 8], there was a scarcity of evidence in patients at low surgical risk [9, 10]. The growing evidence base continues to support the selection of TAVI as a treatment option for an increasingly large group of patients with sSAS [11]. TAVI can now be considered across a spectrum of severity of risk for surgical mortality from those at high risk [12], intermediate risk [13], to patients at low risk [5]. Current European guidelines recommend TAVI in older patients (≥ 75 years), regardless of their surgical risk, if they are suitable for a transfemoral approach (class IA indication: Evidence and/or general agreement that a give treatment or procedure is beneficial, useful and effective, based on evidence from multiple randomized trials or meta-analyses) [14], and advocate that the choice of intervention must be based upon careful evaluation of clinical, anatomical and procedural factors by the Heart Team in discussion with the patient.

TAVI was launched in the Netherlands in 2005. However, despite the consistent findings of multiple landmark randomized trials and guideline recommendations, there is ongoing debate about the added value of TAVI and reimbursement in the Netherlands [12]. In 2020, Zorginstituut Nederland assessed whether TAVI meets the criterion of ‘established medical science and medical practice’ to be eligible for reimbursement. The National Health Care Institute concluded that while TAVI can provide added value for patients with a high surgical risk but for patients at low or intermediate surgical risk, TAVI has not yet been sufficiently proven effective in the longer term (> 2 years) [15]. The cost-effectiveness of TAVI was found to be unfavourable based on two studies that were conducted in inoperable or high-risk patients [16, 17]. More recent evidence, reflecting current practice, has not yet been taken into account in the Netherlands, contrary to other European countries [18–25].

Recently, various analyses based on the same Markov model structure suggested that TAVI is cost-effective versus SAVR in patients at low risk of surgical mortality in France [18], Italy [24], Spain [25], Germany [23], and Belgium [26]. We performed a cost-utility analysis using PARTNER 3 outcomes in combination with cost data from the Netherlands to adapt this model and assess the value of TAVI with SAPIEN 3 versus SAVR in Dutch patients with severe aortic stenosis and low risk of surgical mortality (Society of Thoracic Surgeons [STS] risk of mortality calculation < 4%).

## Methods

### Model structure

**Fig. 1:**
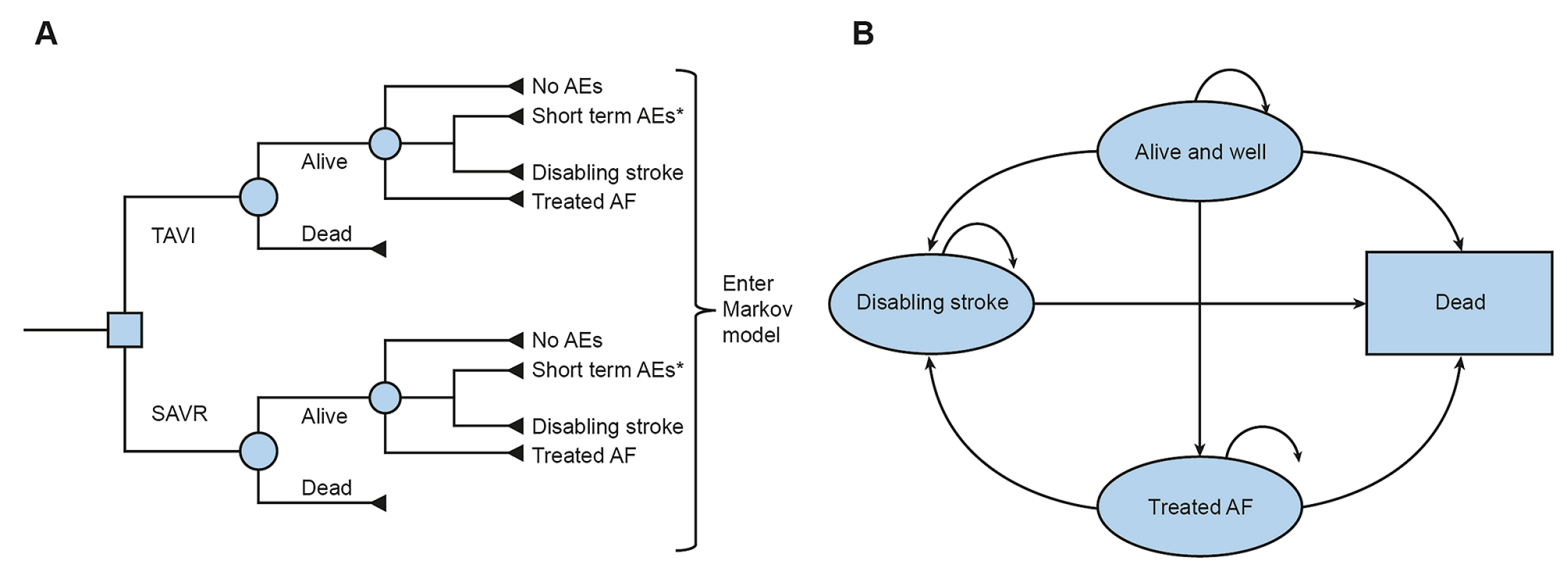
**A)**. 30-day decision tree for short-term AEs* and **B)** four-health-state Markov model to capture longer term outcomes of patients post TAVI or SAVR intervention. *early adverse events (AEs) linked to each procedure were captured using the 30-day AE dataset from the PARTNER 3 trial (2). Transition probabilities between health states were informed by published literature: PARTNER 3 (2) and Evolut low risk [32] trials for ‘alive and well’ to ‘AF’; and a Dutch community-based cohort study (PREVEND) for ‘alive and well’ to ‘disabling stroke’ and ‘AF’ to ‘disabling stroke’ [20]. AE, adverse event; AF, atrial fibrillation; SAVR: surgical aortic valve replacement; TAVI: transcatheter aortic valve implantation

This analysis was carried out to estimate changes in direct healthcare costs, survival, and health-related quality of life (QALYs). A two-stage model was used; early adverse events (AEs) linked to each procedure were captured using the 30-day AE dataset from the PARTNER 3 trial in a decision tree (Fig. 1A) [2]. These data were fed into a Markov model, which included four distinct health states (alive and well; treated atrial fibrillation [AF]; disabling stroke [DS]; and dead) to capture longer-term patient outcomes post TAVI or SAVR intervention (Fig. 1B) [18]. A lifetime horizon (50 years) was used with discounting of future costs and benefits applied at a rate of 4.0% and 1.5%, respectively [27]. Both the conceptual model and input data were validated by the department for Health Evidence of Radboud university medical center (Radboudumc) and a Dutch scientific steering committee.

### Model inputs

#### Clinical events in the cost-effectiveness model

Model inputs were based on the previously published model [18]. Due to the low number of stroke events in PARTNER 3, ‘alive and well’ to ‘DS’ and ‘AF’ to ‘DS’ were captured from a Dutch community-based cohort study (PREVEND) conducted in 8,265 participants in the Netherlands [28]. Transitions to ‘treated AF’ and ‘DS’ were assumed to remain constant over the time horizon from year 2 onwards, regardless of time in the model or patient age. Hospitalization data used in the model were based on 1- and 2-year data from PARTNER 3 and were assumed to remain constant over the time horizon of the model after 2 years. Similarly, the rate of reinterventions was assumed to remain constant after 22 years [29], due to a paucity of longer-term data. In the base case the same reintervention rate was applied to both interventions. However, the impact of increased risk of reintervention with SAPIEN 3 was explored in scenario analyses.

The survival estimates using parametric survival analysis from the 2-year outcomes of the PARTNER 3 trial produced clinically implausible estimates in the model due to a very low rate of death in the study [5, 6]. However, they are included as options in the model and are explored in scenario analyses in combination with general mortality data for the Netherlands to ensure that patients in the model cannot survive for longer than would be expected for the general population.

All-cause mortality in the base case was determined from general population normal mortality risk, with relative risks applied from published literature [30]. Model input probabilities of clinical events are presented in Supplementary Table 1.

#### Utilities

Utility values used age-adjusted population utility norms. An EQ-5D-5 L index value (time trade-odd value set) was used for the age adjusted population utility norms, specific to the Dutch population [31]. A pragmatic search of the literature was conducted to identify disutilities that could be appropriately combined with each health state [31–33]. Health-related quality of life was included in the analysis using quality-adjusted life-years (QALYs) based on the EQ-5D utility values for the different health states in the model, with utility decrements taken from published studies and age-adjusted population norms [31].

#### Cost inputs

Costs were based on costing information from the diagnosis-related groups (DRGs) and published data in intermediate- and high-risk patients [30, 34] (Table 1). Data from Huygens et al. 2018 [35], including rehabilitation, were used as the base case assumption. Costs were expressed in 2020 Euros. Cost correction for inflation was applied when necessary, using the consumer price index from Statistics Netherlands (CBS Statline) [36].

**Table 1:** Costs associated with TAVI and SAVR

Unit cost components	TAVI	SAVR	Source
**Procedure**			
Total cost of initial procedure including rehabilitation	€35,342	€27,902	Huygens et al. 2018
**Acute post-operative complications**			
Re-intervention	€35,342	€35,342	Huygens et al. 2018
**Associated to health states**			
Treated AF - month 1	€108	€108	Verhoef et al. 2014 GIP data bank
Treated AF ≥ month 2	€85	€85	Verhoef et al. 2014 GIP data bank
DS - month 1	€15,784	€15,784	Van Eeden et al. 2015
DS ≥ month 2	€699	€699	
Caregiver for DS - month 1	€244	€244	
Caregiver for DS ≥ month 2	€376	€376	
Alive and well– year 1	€24	€24	Assumed follow up consultation required at months 1, 6 and 12. Cost of outpatient appointment based on OLVG tariff 2014
Alive and well– year 2+	€8	€8	Assumed follow up consultation required once per year. Cost of outpatient appointment based on OLVG tariff 2014
**Other costs considered**			
Pacemaker complications (monthly)	€37	€37	Van Eck et al. 2004 and Udo et al. 2012
Rehospitalization	€2600	€2600	Standaard prijslijst dbc-zorgproducten 2019

AF, atrial fibrillation; DS, disabling stroke; SAVR, surgical aortic valve replacement; TAVI, transcatheter aortic valve implantation

### Model outputs

#### Sensitivity analyses

To evaluate uncertainty in the results, one-way deterministic sensitivity analyses were performed by varying inputs between confidence intervals, where reported or plausible ranges wherein variance data were not available (Supplementary Table 3). Input parameters to which the model’s results are most sensitive were ranked, and the results displayed in terms of incremental net monetary benefit at a cost-effectiveness threshold of €50,000/QALY using a tornado diagram (Supplementary Table 4) [37]. Overall parameter uncertainty was addressed by a probabilistic sensitivity analysis (PSA). Probability distributions for all input parameters were specified and 1000 Monte Carlo simulations were run using random draws of all parameters from within their assigned distributions. Several scenario analyses were performed to examine the impact of major structural assumptions. The model was stretched by running alternative time horizons, discount rates and scenarios, including: the risk of reintervention for those undergoing TAVI based on data from the PARTNER 2 trial [13]; survival and quality of life data from PARTNER 3 [6]; increase in the risk of stroke for those undergoing TAVI; inclusion of adverse event costs for those occurring within 30 days after treatment (considering occurring after discharge from index hospitalization); using various costs assumptions for the procedure; removing impact of disutilities of ‘AF’; and considering simultaneous conservative assumptions.

## Results

### Base case

TAVI generated greater QALYs (incremental improvement of 0.89 per patient) with an increase in costs (incremental cost increase of €4742 per patient) compared with SAVR (Table 2) over a 50-year time horizon. This represents an ICER of €5346 per QALY. Although the initial procedural cost was relatively higher with TAVI, these costs were somewhat offset by lower costs related to ‘DS’ and ‘AF’ health states, compared with SAVR (Supplementary Fig. 1).

**Table 2:** Modelled cost-benefit findings for TAVI compared with SAVR in the Netherlands

Summary results	TAVI	SAVR	Incremental
Cost per patient	**€44,149**	**€39,407**	**€4742**
Life year gained (undiscounted)	12.67	11.89	0.79
Median survival (years)	15.00	13.17	1.83
QALYs per patient	**9.50**	**8.62**	**0.89**
**Incremental cost-effectiveness ratio (ICER)**			**€5346**
**Incremental net monetary benefit (NMB)**			€39,615
**Incremental net health benefit (NHB)**			0.79
**Acute phase cost (initial procedure)**			
**Index hospitalization (without pacemaker)– including rehabilitation**	€36,190	€29,140	€7050
**Acute phase costs**	**€36,190**	**€29,140**	**€7050**
**Additional costs at 1 year**			
MI	€123	€84	€38
Costs of pacemaker complications	€25	€15	€10
Costs of rehospitalizations	€177	€265	–€88
Re-intervention costs	€163	€161	€1
Alive and well health state costs	€270	€181	€89
Treated AF health state costs	€45	€350	–€305
DS costs	€46	€181	–€135
Death costs	€0	€0	€0
**Total costs at 1 year**	**€36,190**	**€29,140**	**€7,050**
**Additional lifetime costs**			
Costs of pacemaker complications	€241	€140	€101
Costs of rehospitalizations	€266	€252	€15
Re-intervention costs	€4,171	€3,642	€529
Alive and well health state costs	€750	€474	€276
Treated AF health state costs	€722	€3075	–€2353
DS costs	€1808	€2684	–€876
Death costs	€0	€0	€0
**Additional lifetime costs**	**€7959**	**€10,267**	**–€2308**
**Total lifetime costs**	**€44,149**	**€39,407**	**€4742**

AF, atrial fibrillation; DS, disabling stroke; ICER, incremental cost-effectiveness ratio; MI, myocardial infarction; NHB, Net health benefit; NMB, net monetary benefit; QALY, quality of life adjusted year; SAVR, surgical aortic valve replacement; TAVI, transcatheter aortic valve implantation

### Deterministic sensitivity analyses

Univariate sensitivity analysis is displayed in a tornado diagram (Supplementary Fig. 2), which shows that TAVI remains cost-effective regardless of changes in individual model parameters. The model is most sensitive to the procedure costs for both TAVI and SAVR and the starting age of patients entering the model.

### Probabilistic sensitivity analysis

**Fig. 2:**
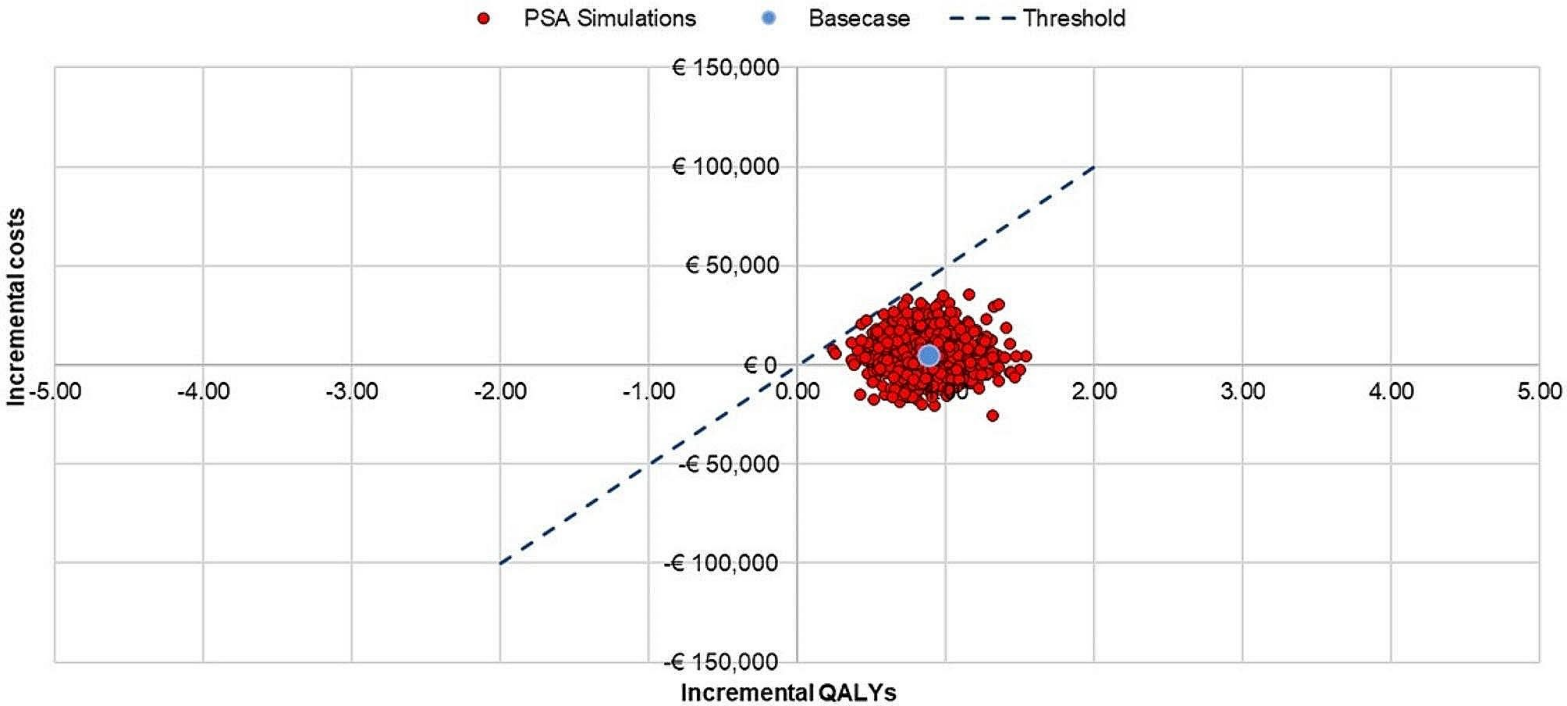
Probablistic sensitivity analysis; all cases in the model fell below a €50,000/QALY threshold. PSA, probabilistic sensitivity analysis; QALY, quality-adjusted life-year

**Fig. 3:**
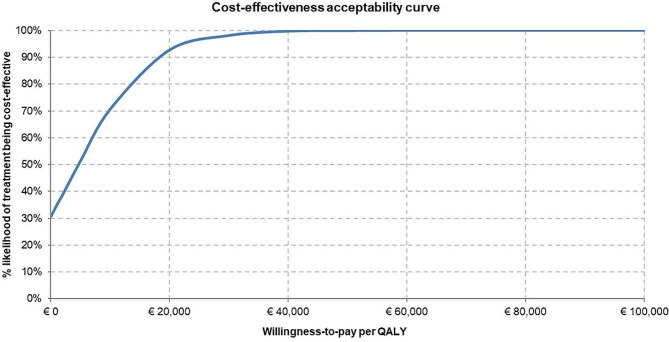
Cost-effectiveness of TAVI across several willingness-to-pay thresholds. QALY, quality-adjusted life-year; SAVR, surgical aortic valve replacement; TAVI, transcatheter aortic valve implantation

The results of the PSA are presented on the cost-effectiveness plane (Fig. 2) and in a cost-effectiveness acceptability curve (Fig. 3). At a threshold of €50,000/QALY or above, TAVI appears to be cost-effective compared with SAVR in 100% of iterations. TAVI is less costly and more effective (dominant) than SAVR in around 29% of iterations and it achieves a 90% cost-effectiveness outcome when the cost-effectiveness threshold is as low as €20,000//QALY.

### Scenarios

A series of scenario analyses were conducted to assess the impact of changing various assumptions on the results of the model. TAVI with SAPIEN 3 remained cost-effective regardless of the time horizon. In other scenarios, SAPIEN 3 TAVI remained cost-effective compared with SAVR, even if AEs costs occurring within 30 days of the procedure were included; survival at 2 years (PARTNER 3 data; HR = 0.75) was adjusted to Dutch general population mortality; a more aggressive reintervention rate for TAVI (based on PARTNER 2 A) was used, including combining this with no survival benefits; an increased rate of stroke with TAVI after 2 years was used; and different estimate for AF disutility (more conservative, null) were used. All scenarios are presented in Supplementary Table 5.

## Discussion

Our analysis shows that TAVI appears to be effective for patients in the Netherlands with aortic stenosis and a low risk of surgical mortality. The benefits for TAVI shown in our model represent a highly cost-effective intervention (ICER=€5346), and these benefits remained robust at a threshold of €50,000/QALY, in multiple scenario analyses. As cost-effectiveness in the Netherlands is based on the maximum reference values/QALY in combination with the burden of disease (threshold between €20,000–80,000) [30], TAVI can be considered cost-effective for this population.

Results of other analyses in low-risk patients support these finding [18–25].

Using the Markov model presented in this paper, TAVI with SAPIEN 3 was shown to be dominant in a recent analysis in France (cost saving of €12,742 and generating greater 0.89 QALYs per patient) [18] and in Belgium (cost saving of €3013 and generating greater 0.94 QALYs per patient) [26]; this model and cost outputs also informed the positive French HTA appraisal made in March 2021 [21]. In Italy and Spain, TAVI with SAPIEN 3 was found to be a cost-effective option versus SAVR with an ICER of €2989/QALY and €6952/QALY, respectively [24, 25]. Cost-effectiveness of TAVI with SAPIEN 3 in sSAS patients at low risk of surgical mortality was shown recently in Germany with an ICER of €12,037/QALY [23].

Two additional cost-effectiveness studies of SAPIEN 3, based on PARTNER 3, have been published, showing that SAPIEN 3 was cost-effective in Canada and Australia with respective ICERs of CAN$27,196/QALY and AUS$3521/QALY gained [38, 39].

### Study limitations

There are inherent limitations to a cost-effectiveness model including assumptions made in the presence of ‘best fit’ data or a paucity of data; extrapolations into time horizons that are modelled beyond the scope of existing input data; and the under- and overestimations potentially caused by differences in healthcare systems, or by the criteria for intervention and treatment selection within that specific system.

The PARTNER 3 study population was largely (97.1%) from the USA, and certain patient characteristics may not translate to the Dutch population. Efforts were made to incorporate Dutch-specific data into the model, including the PREVEND community study [34], adjustment of the EQ-5D-5 L index value to representative Dutch parameters, Dutch-specific risk of mortality with TAVI [30], and country-specific life expectancy by age and sex [40].

Hospitalization data were based on 1- and 2-year data from PARTNER 3; however, the impact of our assumption that this rate remained constant over the time horizon of the model after 2 years is unknown as patients in both treatment arms in the model are at risk of hospitalization. Our assumption that the rate of reinterventions remains constant after 22 years is expected to have very little impact on modelled outcomes because only around 11% of the patients would still be alive in the model, with limited need for reintervention.

There are several limitations that may underestimate the benefit of TAVI. It may be expected that, over time, there would be a greater incidence of stroke in the SAVR arm given the higher incidence of AF compared with TAVI; the assumption of similar stroke incidence between arms in this study may underestimate the cost benefit of TAVI. Transitions to ‘AF’ and ‘DS’ were assumed to remain constant over the time horizon from year 2 onwards, regardless of time in the model or patient age, which may understate transitions between disease states in later years because it is likely that stroke risk will increase with age [41].

The cost analysis was partly informed by data from 2013, and the authors recognise that overall costs will have changed over the past decade, driven by updates in protocols, shorter patient stays in hospital and changes in unit price. Furthermore, costs are expenditures reimbursed by health insurers based on agreements between healthcare providers and insurers and this arrangement takes into account more factors than just device costs [35]. However, we considered most meaningful estimates that could be referenced, and we believe that we took a conservative approach to the analysis, meaning that it is likely that today’s practice would lead to more positive results than those reported here.

There are also limitations that may overestimate the benefits of TAVI. The incidence of AF is based on the PARTNER 3 trial and might not reflect the reality in a Dutch practice environment. Further, the PARTNER 3 data at 5 years were not available at the time of the submission of this manuscript and longer-term follow-up might impact the results. Importantly, compared to recently published data in patients at low risk of surgical mortality who were followed for 5 years, there were no ‘red flags’ in the accuracy of the durability, excess risk of mortality, or adverse event data used in this model [42], which should promote confidence in our results.

A further limitation of this type of modelling study is the lack of inclusion of societal costs or future indirect medical costs.

Our model examined the impact of TAVI from only a healthcare perspective. As the views of patients, their carers or a societal perspective have been mostly lacking in HTA of TAVI, we performed an initial scoping exercise. Based on PARTNER 3 trial data, a scoping literature review and interviews with a caregiver (patient representative), an interventional cardiologist, a geriatrician and a nurse practitioner in the Netherlands, we found indications that TAVI may save productivity costs as patients who received TAVI return to work 4 days earlier than patients who had surgery. PARTNER 3 data also showed that more patients after TAVI were discharged to ‘home or selfcare (routine discharge)’, while in the surgery group, more patients were discharged to a skilled nursing facility or an inpatient rehabilitation facility. Concerns about the invasiveness and recovery period regarding SAVR were expressed in a recent Dutch study emphasizing the impact of the disease on daily functioning [43]. Consistent with European clinical guidelines [14], it is common Dutch practice that the cardiologist and Heart Team makes decisions in consultation with the patient, increasing patient autonomy. On the other hand, there might be unintended consequences (e.g. increased catheterization) that should also be taken into account in a comprehensive HTA [19].

Overall, the findings are meaningful in three ways. First, patients may prefer an intervention associated with lower risk of complication and/or rehospitalization, and with improved recovery rate and quality of life gains compared with SAVR. Second, for the appropriate population of patients with severe symptomatic aortic stenosis, TAVI may become the reference treatment provided there remains at least equipoise in terms of clinical outcome between TAVI and SAVR. Third, from the perspective of the healthcare provider, TAVI, compared with SAVR, may result in shorter hospital stays, lower requirement for general anaesthesia, lower risk of infection and fewer complications. Moreover, it is plausible that the cost-effectiveness associated with TAVI in patients at low risk of surgical mortality will translate to procedures in higher-risk patients where care costs should be expected to be greater, and the margin of cost saving versus SAVR could be increased.

## Conclusions

Using 2-year data from the PARTNER 3 trial, our model estimates that TAVI with SAPIEN 3 might be cost-effective for patients with severe symptomatic aortic stenosis at low surgical risk, with an ICER within the limits set by the Dutch policy makers. It also seems likely that the TAVI procedure provides broader beneficial effects, and these findings could be used to optimize appropriate intervention selection for this patient population in the Netherlands.

### Electronic supplementary material

Below is the link to the electronic supplementary material.

Supplementary Material 1
