# Supplementary material for: Cost-utility analysis of TAVI compared with surgery in patients with severe aortic stenosis at low risk of surgical mortality in the Netherlands
Source: Cost Eff Resour Alloc. 2024 Mar 26;22:24. doi: 10.1186/s12962-024-00531-6 (PMC10964658; doi:10.1186/s12962-024-00531-6)
Supplement: Supplementary file 1 — Supplementary Material 1 [file 12962_2024_531_MOESM1_ESM.docx]

**Supplementary Figures and Tables**

**Supplementary Fig. 1. Lifetime cost distribution**

SAVR, surgical aortic valve replacement; TAVI, transcatheter aortic valve implantation.

**Supplementary Fig. 2.** Tornado diagram of the deterministic sensitivity analyses*.*

*
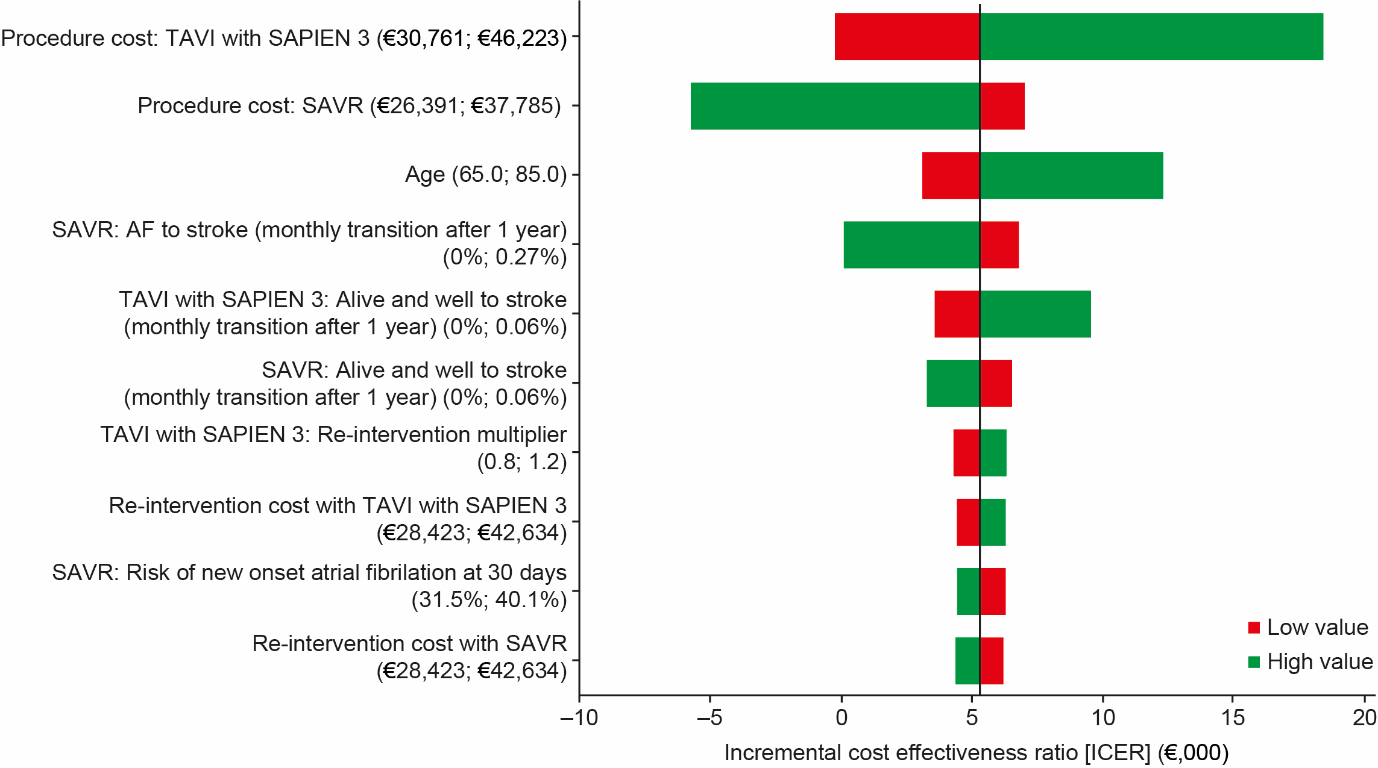
*AF, atrial fibrillation; ICER, incremental cost-effectiveness ratio; SAVR, surgical aortic valve replacement; TAVI, transcatheter aortic valve implantation.

**Supplementary Table 1.** Probabilities of clinical events used in the model.

| **Clinical events** | **TAVI** | **SAVR** | **Source** |
| --- | --- | --- | --- |
| **At 30 days** | | | |
| Treated AF | 4.1% | 35.8% | PARTNER 3 trial [1] |
| New permanent pacemaker | 6.5% | 4.0% | PARTNER 3 trial |
| Rehospitalization | 3.4% | 6.4% | PARTNER 3 trial |
| DS | 0.0% | 0.4% | PARTNER 3 trial |
| Aortic reintervention | 0.2% | 0.7% | PARTNER 3 trial |
| Mortality | 0.4% | 1.1% | PARTNER 3 trial |
| **Monthly health states transition probabilities and intercurrent events between 30 days and 1 year** | | | |
| Alive and well 🡪  Treated AF | 0.11% | 0.15% | PARTNER 3 trial |
| Alive and well 🡪 DS | 0.02% | 0.02% | Vermond *et al.* 2015 [2] |
| Treated AF 🡪  DS | 0.05% | 0.05% | Vermond *et al.* 2015 |
| TIA | 0.09% | 0.04% | PARTNER 3 trial |
| MI | 0.02% | 0.08% | PARTNER 3 trial |
| Severe or Life-threatening bleeding | 0.4% | 0.1% | PARTNER 3 trial |
| **Monthly health states transition probabilities after 1 year and beyond** | | | |
| Alive and well 🡪  Treated AF | 0.06% | 0.11% | PARTNER 3 trial |
| Alive and well 🡪  DS | 0.02% | 0.02% | Vermond *et al.* 2015 |
| Treated AF 🡪  DS | 0.05% | 0.05% | Vermond *et al.* 2015 |
| **Rehospitalization Events (converted to monthly rates in calculations)** | | | |
| Rehospitalization at 1y | 4.2% | 4.8% | PARTNER 3 trial |
| Rehospitalization at 2y and beyond | 1.2% | 1.2% | PARTNER 3 trial 2-year outcomes [3] |
| **Aortic reintervention** (converted to monthly rates in calculations) | | | |
| From Year 1 to Year 23 onwards | From 0.5% to 8.9% | From 0.5% to 8.9% | PARTNER 3 up to 2 years. Then Bourguignon *et al*. 2015 [4] |

AF, atrial fibrillation; DS, disabling stroke; SAVR, surgical aortic valve replacement.

Supplementary Table 2. Annual mortality risk for ’alive and well’ and associated relative risk for other health states.

| **Annual mortality risk - alive and well (by age)** | **Males** | | **Females** | **Monthly probability for TAVI** | **Monthly probability for SAVR** | | **Source** |
| --- | --- | --- | --- | --- | --- | --- | --- |
| 70 years | 1.7% | | 1.2% | 0.13% | 0.13% | | StatLine life expectancy by age & gender [5] |
| 71 years | 2.0% | | 1.3% | 0.15% | 0.15% | |  |
| 72 years | 2.2% | | 1.5% | 0.17% | 0.17% | |  |
| 73 years | 2.5% | | 1.6% | 0.18% | 0.18% | |  |
| 74 years | 2.6% | | 1.7% | 0.20% | 0.20% | |  |
| 75 years | 3.2% | | 2.1% | 0.24% | 0.24% | |  |
| 76 years | 3.6% | | 2.4% | 0.27% | 0.27% | |  |
| 77 years | 3.9% | | 2.5% | 0.30% | 0.30% | |  |
| 78 years | 4.5% | | 2.9% | 0.34% | 0.34% | |  |
| 79 years | 5.0% | | 3.3% | 0.38% | 0.38% | |  |
| 80 years | 5.6% | | 3.6% | 0.43% | 0.43% | |  |
| 81 years | 6.4% | | 4.3% | 0.49% | 0.49% | |  |
| 82 years | 6.9% | | 4.8% | 0.54% | 0.54% | |  |
| 83 years | 8.2% | | 5.4% | 0.63% | 0.63% | |  |
| 84 years | 9.3% | | 6.3% | 0.72% | 0.72% | |  |
| 85 years | 10.1% | | 7.0% | 0.79% | 0.79% | |  |
| 86 years | 11.7% | | 8.5% | 0.94% | 0.94% | |  |
| 87 years | 13.5% | | 9.9% | 1.10% | 1.10% | |  |
| 88 years | 14.9% | | 11.2% | 1.23% | 1.23% | |  |
| 89 years | 17.4% | | 12.6% | 1.43% | 1.43% | |  |
| 90 years | 19.1% | | 14.9% | 1.62% | 1.62% | |  |
| 91 years | 20.8% | | 16.4% | 1.78% | 1.78% | |  |
| 92 years | 23.1% | | 18.3% | 2.01% | 2.01% | |  |
| 93 years | 24.6% | | 20.5% | 2.19% | 2.19% | |  |
| 94 years | 27.9% | | 22.7% | 2.51% | 2.51% | |  |
| 95 years | 31.1% | | 24.3% | 2.81% | 2.81% | |  |
| 96 years | 31.2% | | 27.4% | 2.93% | 2.93% | |  |
| 97 years | 35.1% | | 30.6% | 3.37% | 3.37% | |  |
| 98 years | 38.7% | | 31.7% | 3.72% | 3.72% | |  |
| 99 years | 40.2% | | 38.2% | 4.11% | 4.11% | |  |
| 100 years | 40.2% | | 38.2% | 4.11% | 4.11% | |  |
| **Relative risk of death in hazard ratio - associated to treated AF, DS and re-intervention** | | | | | | | |
| Treated AF | | 1.46 | | | | Odutayo *et al*. 2016 [6] | |
| DS | | 2.3 | | | | Giesler *et al.* 2017 [7] | |
| Aortic re-intervention | | 2 | | | | PARTNER 3 trial. Procedural deaths (0.4%) compared to monthly mortality risk of general mortality for 70–74 years old (0.2%). | |

AF, atrial fibrillation; DS, disabling stroke; SAVR, surgical aortic valve replacement; TAVI, transcatheter aortic valve implantation.

**Supplementary Table 3.** DSAs input parameters.

| **Parameter** | **Base case value** | **Lower value** | **Upper value** | **Lower (%)** | **Upper (%)** |
| --- | --- | --- | --- | --- | --- |
| Age | **73** | 65 | 85 | 11.0% | 16.4% |
| Proportion male | **69%** | 64.8% | 73.2% | 6.1% | 6.1% |
| Discount rate: costs | **4.0%** | 3.0% | 5.0% | 25.0% | 25.0% |
| Discount rate: benefits | **1.5%** | 1.0% | 3.0% | 33.3% | 100.0% |
| TAVI with SAPIEN 3: Mortality risk at 30 days | **0.4%** | 0.00% | 0.97% | 100% | 140.6% |
| TAVI with SAPIEN 3: Risk of new onset of treated AF at 30 days | **4.1%** | 2.30% | 5.86% | 43.6% | 43.7% |
| TAVI with SAPIEN 3: Risk of new PP at 30 days | **6.5%** | 4.24% | 8.66% | 34.3% | 34.2% |
| TAVI with SAPIEN 3: Risk of DS at 30 days | **0.0%** | 0.00% | 1.00% | NA | NA |
| SAVR: Mortality risk at 30 days | **1.1%** | 0.16% | 2.04% | 85.5% | 85.2% |
| SAVR: Risk of new onset treated AF at 30 days | **35.8%** | 31.5% | 40.1% | 11.9% | 12.1% |
| SAVR: Risk of new PP at 30 days | **4.0%** | 2.21% | 5.72% | 44.3% | 44.3% |
| SAVR: Risk of DS at 30 days | **0.4%** | 0.00% | 1.00% | 100.0% | 127.0% |
| TAVI with SAPIEN 3: Alive and well to treated AF (monthly transition after 1 year) | **0.1%** | 0.06% | 0.11% | 5.5% | 76.5% |
| TAVI w. SAPIEN 3: Alive and well to DS (monthly transition after 1 year) | **0.02%** | 0.00% | 0.06% | 100.0% | 197.5% |
| TAVI w. SAPIEN 3: Treated AF to DS (monthly transition after 1 year) | **0.1%** | 0.00% | 0.27% | 100.0% | 485.9% |
| SAVR: Alive and well to treated AF (monthly transition after 1 year) | **0.11%** | 0.10% | 0.15% | 7.5% | 38.7% |
| SAVR: Alive and well to DS (monthly transition after 1 year) | **0.02%** | 0.00% | 0.06% | 100.0% | 197.5% |
| SAVR: Treated AF to DS (monthly transition after 1 year) | **0.05%** | 0.00% | 0.27% | 100.0% | 485.9% |
| TAVI: Rehospitalisation multiplier | **1** | 0.8 | 1.2 | 20.0% | 20.0% |
| SAVR: Rehospitalisation multiplier | **1** | 0.8 | 1.2 | 20.0% | 20.0% |
| TAVI:  Re-intervention multiplier | **1** | 0.8 | 1.2 | 20.0% | 20.0% |
| SAVR: Re-intervention multiplier | **1** | 0.8 | 1.2 | 20.0% | 20.0% |
| RR of death for treated AF | **1.5** | 1.39 | 1.54 | 4.8% | 5.5% |
| RR of death with DS (month 1) | **2.3** | 1.30 | 3.00 | 43.5% | 30.4% |
| RR of death with DS (month 2+) | **2.3** | 1.30 | 3.00 | 43.5% | 30.4% |
| RR of death with reintervention TAVI | **2.2** | 1.76 | 2.64 | 20.4% | 19.3% |
| RR of death with reintervention SAVR | **2.2** | 1.76 | 2.64 | 20.4% | 19.3% |
| HR mortality vs SAVR: TAVI | **0.75** | 0.35 | 1.63 | 53.3% | 117.3% |
| Utility decrement: treated AF | **0.16** | 0.12 | 0.19 | 20.0% | 20.0% |
| Utility decrement: DS | **0.21** | 0.17 | 0.25 | 20.0% | 20.0% |
| Procedure cost: TAVI | **€35,342** | €30,761 | €46,223 | 13.0% | 30.8% |
| Procedure cost: SAVR | **€27,902** | €26,391 | €37,785 | 5.4% | 35.4% |
| AEs cost: TAVI | **€0** | €0 | €311 | NA | NA |
| AEs cost: SAVR | **€0** | €0 | €1281 | NA | NA |
| AF cost: per month from month 2 | **€85** | €68 | €102 | 20.0% | 20.0% |
| DS cost (month 1): per month | **€16,028** | €12,823 | €19,234 | 20.0% | 20.0% |
| DS cost (month 2+): per month | **€1075** | €860 | €1290 | 20.0% | 20.0% |
| Alive and well cost: per month (SAVR 2+) | **€8** | €6 | €10 | 20.0% | 20.0% |
| PP complications (per month) | **€37** | €29 | €44 | 20.0% | 20.0% |
| Hospitalizations cost TAVI | **€2600** | €2080 | €3120 | 20.0% | 20.0% |
| Hospitalizations cost SAVR | **€2600** | €2080 | €3120 | 20.0% | 20.0% |
| Re-intervention cost with TAVI | **€35,342** | €28,423 | €42,634 | 19.6% | 20.6% |
| Re-intervention cost with SAVR | **€35,342** | €28,423 | €42,634 | 19.6% | 20.6% |

AF: atrial fibrillation; DS: disabling stroke; HR: hazard ratio; RR: relative risk; SAVR, surgical aortic valve replacement; TAVI, transcatheter aortic valve implantation

**Supplementary Table 4.** Probabilistic sensitivity analysis assumptions.

| **Parameter** | **Base case value** | **Distribution** | **Parameters** | **Source** |
| --- | --- | --- | --- | --- |
| **TAVI with SAPIEN 3 – clinical events at 30 days** | | | | |
| All-cause mortality | **0.4%** | Beta | Alpha = 2 / Beta = 494 | PARTNER 3 (3) |
| Rehospitalization | **3.4%** | Beta | Alpha = 17 / Beta = 479 | PARTNER 3 |
| Aortic re-intervention | **0.2%** | Beta | Alpha = 1 / Beta = 495 | PARTNER 3 |
| **SAVR – clinical events at 30 days** | | | | |
| All-cause mortality | **1.1%** | Beta | Alpha = 5 / Beta = 449 | PARTNER 3 |
| New onset of treated AF | **35.8%** | Beta | Alpha = 132 / Beta = 237 | PARTNER 3 |
| New permanent pacemaker | **4.0%** | Beta | Alpha = 18 / Beta = 436 | PARTNER 3 |
| Rehospitalization | **6.4%** | Beta | Alpha = 29 / Beta = 425 | PARTNER 3 |
| Aortic re-intervention | **0.7%** | Beta | Alpha = 3 / Beta = 451 | PARTNER 3 |
| **TAVI with SAPIEN 3 – clinical events from 30 days to 1 year (monthly rate)** | | | | |
| TIA | **0.09%** | Beta | Alpha = 5 / Beta = 491 | PARTNER 3 |
| Myocardial infarction | **0.02%** | Beta | Alpha = 1 / Beta = 495 | PARTNER 3 |
| Severe/life-threatening bleeding | **0.4%** | Beta | Alpha = 20 / Beta = 476 | PARTNER 3 |
| Rehospitalization | **0.35%** | Gamma | SD = 0.01 (assumption) | PARTNER 3 |
| **SAVR – Clinical events from 30 days to 1 year (monthly rate)** | | | | |
| TIA | **0.04%** | Beta | Alpha = 2 / Beta = 452 | PARTNER 3 |
| Myocardial infarction | **0.08%** | Beta | Alpha = 4 / Beta = 450 | PARTNER 3 |
| Severe/life-threatening bleeding | **0.1%** | Beta | Alpha = 6 / Beta = 448 | PARTNER 3 |
| Rehospitalization | **0.4%** | Gamma | SD = 0.01 (assumption) | PARTNER 3 |
| **TAVI with SAPIEN 3 – monthly transition probabilities (Month 1 to 12 and Month 13 onwards)** | | | | |
| Alive and well to treated AF – M1 to M12 | **0.11%** | Beta | Alpha = 5 / Beta = 395 | PARTNER 3 |
| Alive and well to treated AF – M13 onwards | **0.06%** | Beta | Alpha = 3 / Beta = 392 | PARTNER 3 |
| Alive and well to DS | **0.02%** | Beta | Alpha=19 / Beta=7981 | Vermond *et al.* 2015 |
| Treated AF to DS | **0.05%** | Beta | Alpha=1 / Beta=264 | Vermond *et al.* 2015 |
| **SAVR – Monthly transition probabilities (Month 1 to 12 and Month 13 onwards)** | | | | |
| Alive and well to treated AF – M1 to M12 | **0.15%** | Beta | Alpha = 4 / Beta = 233 | PARTNER 3 |
| Alive and well to treated AF – M13 Onwards | **0.11%** | Beta | Alpha = 3 / Beta = 230 | PARTNER 3 |
| Alive and well to DS | **0.02%** | Beta | Alpha=19 / Beta=7981 | Vermond *et al.* 2015 |
| Treated AF to DS | **0.05%** | Beta | Alpha=1 / Beta=264 | Vermond *et al.* 2015 |
| **Other clinical events rates for both arms (TAVI and SAVR)** | | | | |
| Rehospitalization – M13 onwards | **1.2%** | Gamma | SD = 0.1 | PARTNER 3 |
| Reintervention rate – Year 1 | **0.5%** | Gamma | SD = 0.05 | PARTNER 3 up to 2y. Then Bourguignon *et al*. Assumption: SD = 10% of mean |
| Reintervention rate – Year 2 | **0.4%** | Gamma | SD = 0.04 |  |
| Reintervention rate – Year 3 | **0.2%** | Gamma | SD = 0.02 |  |
| Reintervention rate – Year 4 | **0.2%** | Gamma | SD = 0.02 |  |
| Reintervention rate – Year 5 | **0.1%** | Gamma | SD = 0.01 |  |
| Reintervention rate – Year 6 | **0.2%** | Gamma | SD = 0.02 |  |
| Reintervention rate – Year 7 | **0.3%** | Gamma | SD = 0.03 |  |
| Reintervention rate – Year 8 | **0.6%** | Gamma | SD = 0.06 |  |
| Reintervention rate – Year 9 | **0.8%** | Gamma | SD = 0.08 |  |
| Reintervention rate – Year 10 | **1.5%** | Gamma | SD = 0.15 |  |
| Reintervention rate – Year 11 | **1.8%** | Gamma | SD = 0.18 |  |
| Reintervention rate – Year 12 | **2.4%** | Gamma | SD = 0.24 |  |
| Reintervention rate – Year 13 | **2.7%** | Gamma | SD = 0.27 |  |
| Reintervention rate – Year 14 | **3.2%** | Gamma | SD = 0.32 |  |
| Reintervention rate – Year 15 | **3.9%** | Gamma | SD = 0.39 |  |
| Reintervention rate – Year 16 | **4.6%** | Gamma | SD = 0.46 |  |
| Reintervention rate – Year 17 | **5.5%** | Gamma | SD = 0.55 |  |
| Reintervention rate – Year 18 | **6.2%** | Gamma | SD = 0.62 |  |
| Reintervention rate – Year 19 | **7.2%** | Gamma | SD = 0.72 |  |
| Reintervention rate – Year 20 | **7.6%** | Gamma | SD = 0.76 |  |
| Reintervention rate – Year 21 | **7.9%** | Gamma | SD = 0.79 |  |
| Reintervention rate – Year 22 | **8.6%** | Gamma | SD = 0.86 |  |
| Reintervention rate – Year 23 onwards | **8.9%** | Gamma | SD = 0.89 |  |
| **Relative risk of death in hazard ratio - associated to treated AF, DS and re-intervention – for both arms** | | | | |
| Treated AF | **1.46** | Lognormal | SD = 0.146 | SD = 10% of mean |
| DS | **2.3** | Lognormal | SD = 0.23 | SD = 10% of mean |
| Aortic reintervention | **2.2** | Lognormal | SD = 0.22 | SD = 10% of mean |
| **Disutilities associated to treated AF and DS– for both arms** | | | | |
| Utility decrement: treated AF | **0.16** | Gamma | SD = 0.02 | SD = 10% of mean |
| Utility decrement: DS | **0.21** | Gamma | SD = 0.02 | SD = 10% of mean |
| **Cost of the procedure (incl. rehab)** | | | | |
| TAVI | **€ 35 342** | Gamma | SD = 7 068 | SD = 20% of mean |
| SAVR | **€ 27 902** | Gamma | SD = 5 580 | SD = 20% of mean |
| **Cost of post-operative complications – for both arms** | | | | |
| Reintervention with TAVI | **€ 35 342** | Gamma | SD = 7 068 | SD = 20% of mean |
| **Monthly cost associated to health states (Alive & well, treated AF, DS)** | | | | |
| Treated AF up to 30 days | **€ 107** | Gamma | SD = 10.8 | SD = 10% of mean |
| Treated AF – M2 onwards | **€ 85** | Gamma | SD = 8.5 | SD = 10% of mean |
| DS (including caregiver) up to 30 days | **€ 16 028** | Gamma | SD = 1 578 | SD = 10% of mean |
| DS (including caregiver) – M2 onwards | **€ 1 075** | Gamma | SD = 70 | SD = 10% of mean |
| Alive and well up to 1 year | **€ 24** | Gamma | SD = 2.4 | SD = 10% of mean |
| Alive and well – 13M onwards | **€ 8** | Gamma | SD = 0.8 | SD = 10% of mean |
| **Other costs considered in the model – for both arms** | | | | |
| Monthly cost of permanent pacemaker | **€ 37** | Gamma | SD = 4 | SD = 10% of mean |
| Rehospitalization | **€ 2 600** | Gamma | SD = 260 | SD = 10% of mean |

AF: atrial fibrillation; DS: disabling stroke; TAVI: transcatheter aortic valve implantation; TIA: transient ischemic attack; SAVR: surgical aortic valve replacement; SD: standard deviation

**Supplementary Table 5.** Scenario analyses results

| **Scenario** | **Cost difference (TAVI vs SAVR)** | **QALY difference (TAVI vs SAVR)** | **ICER** |
| --- | --- | --- | --- |
| Base case | €4742 | 0.89 | €5346 |
| Increase in risk of reintervention with TAVI (RR from Partner 2A) | €13,879 | 0.87 | €15,973 |
| Survival data from Partner 3, 2-year HR 0.75 | €5579 | 1.62 | €3437 |
| No survival benefit (HR 1) | €3335 | 0.47 | €7159 |
| Use of health-related quality of life data by treatment from Partner 3 | €4742 | 0.46 | €10,317 |
| Increase in risk of stroke to align with Partner 3 outcomes | €6470 | 0.93 | €6958 |
| Including adverse event costs within 30 days | €3930 | 0.89 | €4430 |
| Alternative cost estimates of procedure cost | €2506 to €5904 | 0.89 | €2825 to €6655 |
| 5-year time horizon | €5860 | 0.21 | €27,815 |
| 10-year time horizon | €4869 | 0.44 | €10,962 |
| 15-year time horizon | €4524 | 0.67 | €6765 |
| 20-year time horizon | €4623 | 0.82 | €5623 |
| More conservative disutility for AF (0.109, weighted average of EHRA 1-4) | €4742 | 0.90 | €5274 |
| Disutility for AF = 0 | €4742 | 0.57 | €8336 |
| Disutility for AF 0, RR of death for AF = 1 | €3777 | 0.13 | €29,956 |
| Same transition probability to AF after 30 days between treatment arms (both equal to TAVI) | €4869 | 0.95 | €5122 |
| Same AF incidence between arms (including 30-day data) Assumed equal to TAVI | €7115 | 0.08 | €84,115 |
| HR for mortality = 1 (using P3 mortality data) and disutility for AF = 0 | €3335 | 0.10 | €34,720 |

AF: atrial fibrillation; HR: hazard ratio; RR: relative risk; TAVI: transcatheter aortic valve implantation; SAVR: surgical aortic valve replacement.

**References**

1. Mack MJ, Leon MB, Thourani VH, et al. Transcatheter Aortic-Valve Replacement with a Balloon-Expandable Valve in Low-Risk Patients. N Engl J Med. 2019;380:1695-705.

2. Vermond RA, Geelhoed B, Verweij N, et al. Incidence of Atrial Fibrillation and Relationship With Cardiovascular Events, Heart Failure, and Mortality: A Community-Based Study From the Netherlands. J Am Coll Cardiol. 2015;66:1000-7.

3. Leon MB, Mack MJ, Hahn RT, et al. Outcomes 2 Years After Transcatheter Aortic Valve Replacement in Patients at Low Surgical Risk. J Am Coll Cardiol. 2021;77:1149-61.

4. Bourguignon T, Bouquiaux-Stablo AL, Candolfi P, et al. Very long-term outcomes of the Carpentier-Edwards Perimount valve in aortic position. Ann Thorac Surg. 2015;99:831-7.

5. Statline. Statistics Netherlands. 2022. Available from: https://opendata.cbs.nl/statline/#/CBS/nl/dataset/37360ned/table?froms.

6. Odutayo A, Wong CX, Hsiao AJ, Hopewell S, Altman DG, Emdin CA. Atrial fibrillation and risks of cardiovascular disease, renal disease, and death: systematic review and meta-analysis. BMJ. 2016;354:i4482.

7. Geisler B, Huygens S, Reardon M, et al. Cost-Effectiveness and Projected Survival of Self-Expanding Transcatheter Versus Surgical Aortic Valve Replacement for High Risk Patients in a European Setting: A Dutch Analysis Based on the CoreValve High Risk Trial. Structural Heart. 2017;1:267–74.
